# Genome-wide analysis of the WSD family in sunflower and functional identification of *HaWSD9* involvement in wax ester biosynthesis and osmotic stress

**DOI:** 10.3389/fpls.2022.975853

**Published:** 2022-09-23

**Authors:** Cheng Zhang, Jiabao Yang, Wanqiu Meng, Linglu Zeng, Li Sun

**Affiliations:** College of Life Sciences, Shihezi University, Shihezi, China

**Keywords:** *Helianthus annuus* L., wax ester synthase/diacylglycerol acyltransferase, cuticular wax, abiotic stress, transgenic Arabidopsis

## Abstract

The wax esters are important cuticular wax composition that cover the outer surface of plant organs and play a critical role in protection and energy metabolism. Wax ester synthesis in plant is catalyzed by a bifunctional wax ester synthase/acyl-CoA: diacylglycerol acyltransferase (WSD). Sunflower (*Helianthus annuus* L.) is an important oil crop in the world; however, little is known about WSD in sunflower. In this study, we identified and performed a functional analysis of twelve *HaWSD* genes from sunflower genome. Tissue-specific expression revealed that 12 *HaWSD* genes were differentially expressed in various organs and tissues of sunflower, except seeds. *HaWSD* genes were highly induced by salinity, drought, cold, and abscisic acid (ABA) in sunflower. To ascertain their function, *HaWSD9*, with highly expressed levels in stems and leaves, was cloned and expressed in a yeast mutant defective in triacylglycerol (TAG) biosynthesis. *HaWSD9* complemented the phenotype by producing wax ester but not TAG *in vivo*, indicating that it functions as a wax ester synthase. Subcellular localization analysis indicated that HaWSD9 was located in the endoplasmic reticulum (ER). Heterologous introduction of *HaWSD9* into Arabidopsis *wsd1* mutant exhibited increased epicuticular wax crystals and cuticular wax contents on the stems. As compared with the *wsd1* mutant, *HaWSD9* overexpressing transgenic Arabidopsis showed less cuticle permeability, chlorophyll leaching and water loss rate. Further analysis showed that the *HaWSD9* transgenics enhanced tolerance to ABA, mannitol, drought and salinity, and maintained higher leaf relative water content (RWC) than the *wsd1* mutant under drought stress, suggesting that *HaWSD9* play an important physiological role in stress response as well as wax synthase. These results contribute to understanding the function of *HaWSD* genes in wax ester synthesis and stress tolerance in sunflower.

## Introduction

In terrestrial plants, the aerial epidermis is covered with a hydrophobic layer called the cuticle, which is composed of cutin polyester matrix, epicuticular wax, and intracuticular wax, providing protection to plants against environmental stresses (Samuels et al., 2008). The outermost layer of the cuticle, the epicuticular wax, either as a thin film or as diversely shaped wax crystals that give plants the white-bluish appearance called glaucousness (Koch and Ensikat, 2008). The cuticular waxes in plant consist of complex mixtures of hydrophobic lipids, including very-long-chain fatty acids (VLCFAs, C20-C34 chains), primary and secondary alcohols, alkanes, aldehydes, ketones, and wax esters with a small portion of phenylpropanoids and triterpenoids (Kunst and Samuels, 2003; Kunst and Samuels, 2009). Due to their physical and chemical properties, cuticular waxes are useful in protecting plants against abiotic and biotic stresses. For example, it can control non-stomatal water loss on leaf surface, allowing gas exchange and residual transpiration (Riederer and Schreiber, 2001; Hasanuzzaman et al., 2017), protecting plants from UV radiation (Krauss et al., 1997; Solovchenko and Merzlyak, 2003), and control plant-insects interactions, fungal, and bacterial pathogens (Lee and Suh, 2015a).

Cuticular waxes are produced in the surfaces of epidermal cells (Suh et al., 2005), where C16 or C18 fatty acids are synthesized in plastids and further elongated in the endoplasmic reticulum (ER) membrane by fatty acid elongase (FAE) complex (with carbon length up to >34) to form VLCFAs (Kunst and Samuels, 2009). The VLCFAs are converted into VLCFA-CoAs by long chain acyl-CoA synthetase (LACS) (Lü et al., 2009), and then divided into VLCFA derivatives in the ER by the alkane- or alcohol-forming pathway (Kunst and Samuels, 2009). In the alkane-forming pathway, VLCFAs are catalyzed into alkanes by ECERIFERUM 1 (CER1), CER3, and cytochrome b5 (CYTB5) complex (Bernard et al., 2012), and the alkanes are oxidized into ketones and secondary alcohols by mid-chain alkane hydroxylase (MAH1) (Greer et al., 2007). In the alcohol-forming pathway, VLCFAs are reduced into primary alcohols by fatty acyl-CoA reductases (FARs) (Rowland et al., 2006). Primary alcohols are further esterified with fatty acids to form wax esters by bifunctional wax synthase/acyl-CoA: diacylglycerol acyltransferase (WS/DGAT or WSD) (Li et al., 2008).

DGAT (EC 3.2.1.20) enzyme is encoded by different genes, which is considered as a key rate-limiting enzyme catalyzing the conversion of diacylglycerol (DAG) into TAG in plant. Based on structure and activity, DGATs have been divided into four classes: DGAT1, DGAT2, DGAT3 and WS/DGAT. Among them, the well-characterized DGATs in plants are DGAT1 and DGAT2. Although both DGAT and WS/DGAT enzymes have acyltransferase activities, they shared little protein sequence similarities each other (Zhang et al., 2017). The first WS/DGAT enzyme was identified from *Acinetobacter calcoaceticus* and had activity of both DGAT and wax ester synthase (Kalscheuer and Steinbüchel, 2003). The first plant wax ester synthase, PhWS1, which shares sequence similarity with Acinetobacter WS/DGAT, was isolated from petunia (King et al., 2007). Heterologous expression of *PhWS1* in TAG-deficient yeast mutant resulted in wax esters production, but failed to restore TAG biosynthesis (King et al., 2007). There are 11 *WS/DGAT* genes in Arabidopsis genome, *AtWSD1* to *AtWSD11* (Li et al., 2008), but their roles in wax ester production have not been elucidated completely (Patwari et al., 2019). *AtWSD1* (AT5G37300) gene has been studied to predominantly catalyze wax ester formation in Arabidopsis, and it exhibited a high level of wax ester synthase activity, but 10-fold lower DGAT activity when expressed in *Escherichia coli* (Li et al., 2008). *wsd1* mutants resulted in the reduction of wax ester load in Arabidopsis leaf and stem, while the amounts of wax ester in *wsd6* or *wsd7* mutants was not significantly changed (Patwari et al., 2019). Even though remarkable advances have been made in studying *WS/DGAT* genes in Arabidopsis, which emphasize the importance of these enzymes in the accumulation of wax esters, little is known about their function in most other plant species.

Plant have evolved complex structural, physiological and biochemical mechanisms to cope with abiotic stresses. Under drought stress, plant have developed defense strategies to prevent water loss by increasing cuticular wax accumulation. Under drought stress, the total content of cuticular wax increased by 2-3-fold in Arabidopsis and tree tobacco (*Nicotiana glauca*) (Cameron et al., 2006; Kosma et al., 2009). Until now, WSDs are the least characterized enzymes among four DGAT families in plants. Drought, NaCl, and abscisic acid (ABA) strongly upregulated the expression of *AtWSD1* transcripts. Overexpression of *AtWSD1* in Arabidopsis and *Camelina sativa* increased cuticle waxes and enhanced drought tolerance (Abdullah et al., 2021). Furthermore, Arabidopsis transcription factors *AtMYB94* and *AtMYB96* are involved in the biosynthesis of cuticular wax, and the expression level of *AtWSD1* was up regulated under drought treatment (Seo et al., 2011; Lee and Suh, 2015b; Lee et al., 2016).

Sunflower (*Helianthus annuus* L.) is considered the fourth most important oilseed crops in the world and is mainly cultivated in the arid and semiarid regions of northern China (Li et al., 2009). During their lifecycle, sunflower plants are often subjected to various abiotic/biotic stresses, especially in northern China where sunflower is mainly cultivated. Among them, drought stress is one of the most significant limiting factors that affect sunflower development and production (Fulda et al., 2011). Therefore, identification of sunflower stress tolerance genes has great significance in the elucidation of abiotic-stress tolerance pathways and maintain sustainable sunflower production. Although several studies have elucidated the important role of cuticular waxes in plant resistance to environmental stresses, little is known about the wax-related genes in regulating wax biosynthesis in sunflower. In this study, *HaWSD* genes were searched and identified from sunflower genome database, and their expression patterns in different tissues and under various stresses (drought, salt, cold and ABA) were analyzed using quantitative real-time PCR (qRT-PCR). Then, the function of *HaWSD9* in wax ester synthesis and drought tolerance was identified by heterologous expression in the yeast *Saccharomyces cerevisiae* and Arabidopsis mutant. This study provides an important insight into the molecular mechanisms of cuticular wax synthesis and drought stress in sunflower.

## Materials and methods

### Identification of *HaWSD* genes in sunflower genome

The genome and protein sequences data of sunflower was obtained from Ensembl database (http://plants.ensembl.org/index.html). To identify sunflower *HaWSD* candidates, the Hidden Markov Model (HMM) profiles of PF03982 (DGAT domain) was obtained from the Pfam protein family database (http://pfam.xfam.org). Arabidopsis *WSD1* (At5g37300) sequence was utilized as the query to search sunflower *WSD* genes with HMMER (https://www.ebi.ac.uk/Tools/hmmer/). To ensure the integrity of HaWSD members, Arabidopsis *WSD* gene sequences from TAIR (https://www.arabidopsis.org/) were used as queries in BLASTp searches in the sunflower genome database. After removal of redundant sequences, the putative HaWSD protein sequences were submitted to websites SMART and NCBI-CDD for reconfirmation. Correspondingly, the isoelectric point (pI) and molecular weight (MW) of HaWSD proteins were predicted through ExPasy (http://web.expasy.org/). The diagram of functional domains of HaWSD proteins were predicted by My Domain tools (http://prosite.expasy.org/mydomains/).

### Phylogenetic analysis and chromosomal location of *HaWSD* genes

Alignment of HaWSD proteins were carried out using Clustal W program (Thompson et al., 1994). The phylogenetic tree was constructed using the neighbor-joining (NJ) method in MEGA 7 software with 1,000 bootstrap replicates. The location and distribution of *HaWSD* genes were visualized on the chromosomes using the MapGene2Chrom web v2 software.

### Plant materials and growth conditions

Sunflower cultivar T303 is a widely cultivated in the northwest of China with high yield and wild adaptability. Therefore, we selected this cultivar to investigate the expressions of *HaWSD* genes in various tissues and under various abiotic stresses. Seeds were grown in an experimental field in Shihezi University, Xinjiang, China. For tissue-specific analysis, vegetable tissues (roots, stems, cotyledons and leaves) were collected from 4-week-old sunflower seedlings. Sunflower heads were bagged with nylon mesh before anthesis, and bracts, corollas as well as seed embryos at different developmental stages were collected at 7 d intervals from 10 to 38 DAF (days after flowering).

The Arabidopsis T-DNA insertion mutants *wsd1* (SALK_123272C), Columbia ecotype (Col-0), was ordered from NASC (the Nottingham Arabidopsis Stock Centre). The T-DNA insertion site is located in the 5’-untranslated region (UTR) of the *AtWSD1* gene (At5g37300), 400 bp upstream from the transcription initiation site (**Supplementary Figure S1A**). The homozygous mutant lines were identified by PCR of genome DNA with LP, RP and LB primers (**Supplementary Table S1**; **Supplementary Figure S1B**) according to the method described by the Salk Institute Genomic Analysis Laboratory (http://signal.salk.edu/tdnaprimers.2.html). *AtWSD1* mRNA was identified through semi-quantitative RT-PCR in leaves of *wsd1* mutant, indicating that the mRNA levels of *AtWSD1* were reduced in *wsd1* mutant compared with wild type (Col-0) (**Supplementary Figure S1C**). Arabidopsis plants were grown in a growth chamber at 22-24°C, a 16 h light/8 h dark photoperiod, light intensity of 150 μmol photons m^−2^ s^−1^. Wild-type Arabidopsis plants, ecotype Columbia, was used as control.

### Stress treatments of sunflower

Sterilized sunflower T303 seeds were sowed in plastic pots (20 cm×20 cm) containing fertile soil mix with vermiculite (1:1, v/v). Plants were grown in 16 h light/8 h dark photoperiod at 23-25°C (day/night), light intensity of 800 μmol m^−2^ s^−1^, and 55-60% relative humidity. Four-week-old seedlings of sunflower were used for cold, drought, salt and ABA treatments. For cold stress, the seedlings were cultivated in an artificial climate chamber at 4°C. For drought stress, the plants were treated with 15% PEG 6000 to simulate water deficit (Li et al., 2020). For salt stress, the seedlings were exposed to 100 mM NaCl. To test the effects of exogenous ABA treatment, seedlings were treated with exogenous ABA (100 μM) or sterile distilled water (control). Roots, stems and leaves for each treatment were collected from 0, 12, or 24 h after stress treatment.

### RNA extraction and quantitative RT-PCR analysis

Total RNA was extracted with an OminiPlant RNA Kit (CWBIO, Beijing, China) according to the manufacturer's protocols, and cDNA was synthesized with PrimeScript™ reverse transcriptase (TaKaRa, Dalian, China). Primer Premier 5 was used to design primers specific to the *HaWSD9* gene and sunflower 18s rRNA gene (AF1057577) was used as an internal control (**Supplementary Table S1**). qRT-PCR was performed using a LightCycler 480 (Roche, USA) systems. Each experiment was performed with three biological replicates. Relative expression levels for each gene were calculated using the relative 2^−ΔΔCT^ method (Livak and Schmittgen, 2001), and heatmaps were generated by ImageGP (http://www.ehbio.com/ImageGP).

### Cloning and subcellular localization of *HaWSD9*

Specific primers P1 were used to clone the coding sequence of *HaWSD9* (**Supplementary Table S1**). The PCR products were subcloned to pMD19-T vector (Sangon, Shanghai, China) and sequenced. For subcellular localization of HaWSD9, specific primers P2 (**Supplementary Table S1**) were designed to amplify the full-length ORF sequence of *HaWSD9* without stop codon. The PCR products were transformed into the expression vector pAN580-GFP carrying the CaMV 35S promoter and expressing GFP (Chiu et al., 1996). The resulting recombinant plasmid was designed as pAN580-HaWSD*9*-GFP. The empty vector pAN580-GFP was served as a control. The GFP fusions and an ER marker fusion (HDEL-RFP) were co-transformed into Arabidopsis protoplasts according to established protocol (Nelson et al., 2007). The fluorescence images were observed by fluorescence microscopy (Nikon. Tokyo, Japan).

### Heterologous expression of *HaWSD9* in yeast

The *HaWSD9* gene was amplified by PCR using primers P3 (**Supplementary Table S1**) with *Hind*III and *Xba*I restriction sites. The PCR amplicons were transformed into the yeast shuttle vector pYES2 (Invitrogen, Carlsbad, USA) under the control of galactose-inducible promoter *GAL1*. The plasmid was transformed into a *S. cerevisiae* mutant strain H1246. This strain lacks four acyltransferase genes (*DGA1*, *LRO1*, *ARE1* and *ARE2*) and is deficient in TAG synthesis (Sandager et al., 2002). Yeast transformants were selected on SC-Ura plate (synthetic complete medium lacking uracil), supplemented with 2% (w/v) glucose. After 3 days at 30°C, positive transformants were cultivated in SC-Ura liquid medium containing 2% (w/v) galactose and 2% (w/v) raffinose at 30°C for 48 h. The empty pYES2 vector was transformed into yeast cells as a negative control. Palmitic acid (C16:0) and hexadecanol (C16 alcohol) (final concentration 0.1%, w/v) were dissolved with ethanol and added to the medium as substances.

For total lipid analysis, lipids were extracted from yeast cells following the Bligh and Dyer (1959) method. Thin-layer chromatography (TLC) was performed using hexane-diethylether-acetic acid (70:30:1, v/v/v) and visualized using primuline. Palmityl palmitate (Sigma-Aldrich) and lipid standards mixture (ZZStandard, China) were used as wax ester and TAG reference standards, respectively. Wax esters were examined using gas chromatography-mass spectrometry (GC-MS).

### Expression of *HaWSD9* gene in Arabidopsis

The cDNA of *HaWSD9* was inserted into the pCAMBIA2300 vector downstream of the 35S promoter using the primers P4 with *Sma*I and *BamH*I restriction sites. The constructed pCAMBIA2300-*HaWSD9* was transformed into Agrobacterium GV3101 and introduced into the Arabidopsis *wsd1* mutant seedlings using floral-dip method (Clough and Bent, 1998). The transgenic lines were selected based on kanamycin resistance (50 mg/L) and verified by genomic PCR using specific primers P1. T_2_ transgenic lines were selected by semi-quantitative RT-PCR using primers P5, and two lines with high over-expressions of *HaWSD9* were used for further analysis. Arabidopsis *Actin2* (At3g18780) was used as an internal control using primers P6. All primers used in this study are detailed in **Supplementary Table S1**. The positive homozygous seeds of T_2_ generation were used for further experiments.

### Scanning electron microscopic analysis

The scanning electron microscopy (SEM) was used to examine the pattern of epicuticular wax crystal on leaf and stem surface of Arabidopsis. The leaves and stems were detached from 6-week-old of WT, *wsd1* mutant and the *HaWSD9* transgenic lines and dried as previously described (Mao et al., 2012), and SEM observations were performed by scanning electron microscopy (JSM-6610lv, JEOL).

### Toluidine blue staining, leaf water loss and chlorophyll leaching analyses

Leaves and inflorescences from 6-week-old Arabidopsis were stained with a 0.05% toluidine blue (TB) solution for 10 min, and then rinsed with deionized water for 2–3 times. For the leaf water loss assay, rosette leaves were detached, weighed and measured at the indicated time points. For chlorophyll leaking assay, the detached leaves were weighed and immersed in 80% ethanol. The extraction was conducted by shaking at 25°C in the dark, and their chlorophyll leaking content was detected as described previously (Lolle et al., 1998).

### Transgenic Arabidopsis growth assays for mannitol, ABA, drought, and salt tolerance

Abiotic stress tolerance assays were performed with reference to the methods described previously (Dixit et al., 2018). The WT, *wsd1* and *HaWSD9* transgenic plant seeds were germinated on 1/2 MS agar plates with 1% sucrose in the presence or absence of NaCl (75 mM), D-mannitol (100 mM) or ABA (1 μM), and the seedlings were cultivated in a growth chamber at 22 ± 2°C in 16 h light/8 h dark photoperiod. Twenty-one days after treatment, plants were photographed, and their root length and shoot fresh weight were measured.

To assay of drought stress in soil, 4-week-old Arabidopsis seedlings of WT, *wsd1* and *HaWSD9* transgenic plants were exposed to drought stress for 10 d after seedlings were fully watered. Under control (well-watered) and drought conditions, leaf relative water contents (RWCs) of the *HaWSD9* transgenic plants together with *wsd1* and WT plants were detected according to the method of Patwari et al. (2019).

### Cuticular wax extraction and GC-MS analysis

Cuticular waxes of Arabidopsis stem were extracted in chloroform for 30 s, followed by the addition of 10 μL (1 μg/μL) n-tetracosane as an internal standard. The wax extracts were dried in a nitrogen stream and derivatized with a mixture of 200 μL bis-N, N-(trimethylsilyl) trifluoroacetamide (BSFTA) and 200 μL pyridine at 70°C for 45 min. Samples were concentrated under nitrogen and redissolved in chloroform for GC-MS analysis. The wax monomers were identified based on their electron ionization mass spectrometry spectra. Three biological replicates were used for each sample.

### Statistical analysis

The data were analyzed using the SPSS Statistics software. For the comparison of multiple means, the Duncan’s multiple range tests and Student’s t-test were performed, and P < 0.05 was considered to be significant.

## Results

### Identification and characterization of *HaWSD* from sunflower

A total of 12 WSD-encoding genes were identified in the sunflower genome through BLAST search of the database and named according to their chromosome locations. Details of the *HaWSD* gene family, including gene IDs, locations, length of coding sequences, and protein characteristics are provided in **Table 1**. These 12 putative HaWSD genes encoded proteins ranged from 455 amino acids to 666 amino acids. The molecular weight (MW) and isoelectric point (PI) of HaWSD proteins ranged from 50.68 kDa to 74.73 kDa and from 6.84 to 8.62, respectively. One transmembrane domain (TM) was identified in HaWSD7, HaWSD8 and HaWSD9; whereas, other HaWSD proteins lack the transmembrane domain (**Table 1** **)**.

**Table 1:** *HaWSD* genes identified in the sunflower genome.

Gene Name	ID	Chr.	CDS length (bp)	Length (AA)	pI	MW	TM
HaWSD1	OTG29248	4	2001	666	6.84	74.73	0
HaWSD2	OTG29986	4	1368	455	8.62	50.68	0
HaWSD3	OTG23138	6	1431	476	8.95	54.00	0
HaWSD4	OTG23434	6	1461	486	9.20	55.54	0
HaWSD5	OTG11715	10	1425	474	8.55	52.63	0
HaWSD6	OTG11716	10	1386	461	8.77	51.91	0
HaWSD7	OTG12880	10	1476	491	9.00	56.20	1
HaWSD8	OTG03949	12	1527	508	9.21	57.33	1
HaWSD9	OTG03950	12	1530	509	9.21	57.11	1
HaWSD10	OTF91362	16	1377	458	8.41	51.51	0
HaWSD11	OTF91364	16	1371	456	8.65	50.79	0
HaWSD12	OTF86778	17	1428	475	7.11	53.22	0

Chr., Chromosome; PI, isoelectric point; MW, molecular weight; TM, transmembrane domain.

**Figure 1:**
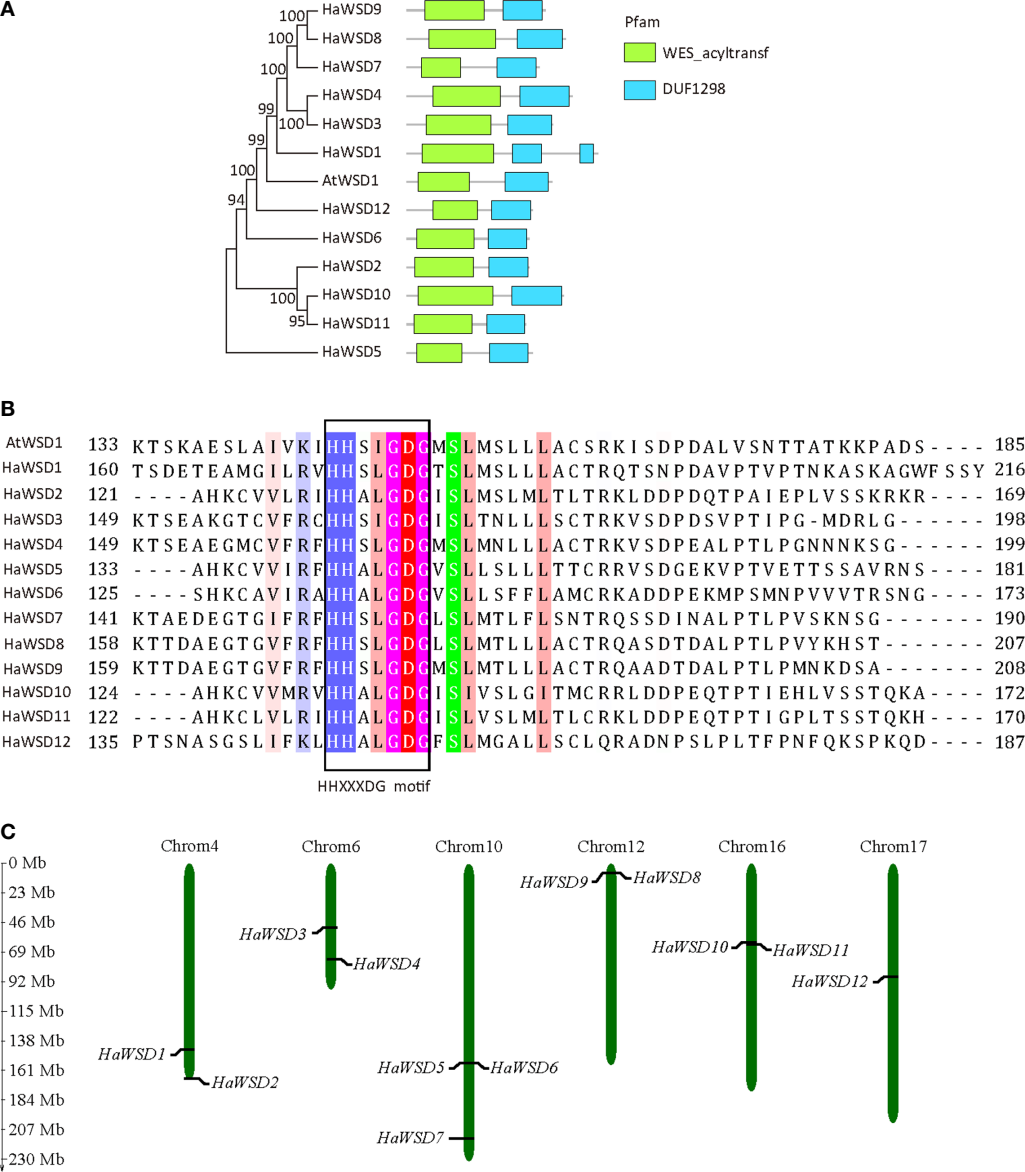
Primary structure and chromosome distributions of HaWSD proteins in sunflower. **(A)** Phylogenetic relationships and conserved domains of the deduced amino acid sequences of HaWSD proteins. The phylogenetic tree was constructed by the neighbor-joining (NJ) method with 1000 bootstrap replicates. The conserved wax ester synthase-like acyl-CoA acyltransferase domain (WES_acyltransf) and DUF1298 domain with unknown function were shown by green and blue squares. **(B)** Amino acids sequences of the putative catalytic site motif HHXXXDG were boxed. **(C)** Chromosome distributions of *HaWSD* genes in sunflower.

Comparisons of deduced amino acid sequences revealed that HaWSDs showed low degree of similarity with Arabidopsis AtWSD1 (23.9% –39.6% identity) (**Supplementary Table S2** **;** **Supplementary Figure S2**). However, All HaWSD proteins contained two domains, a wax ester synthase-like Acyl-CoA acyltransferase domain (WES_acyltransf) and a DUF1298 domain of unknown function, which is similar to AtWSD1 (**Figure 1A**). In addition, all HaWSD proteins contained a conserved acyltransferase active-site motif (HHXXXDG) **(Figure 1B**), which was considered necessary for the catalytic activity of acyl CoA acyltransferase reaction of the synthesis of TAG and wax ester (Kalscheuer and Steinbüchel, 2003), implying that they belong to *WSD* family genes. Chromosomal mapping analysis showed that 12 *HaWSDs* distributed on six sunflower chromosomes. Among them, 3 *HaWSD* genes were identified on chromosome 10. Four chromosomes, 04, 06, 12 and 16 each harbored 2 *HaWSD* genes, while chromosome 17 had just one *HaWSD* gene (**Figure 1C**).

### Tissue expression profile of *HaWSD* genes

**Figure 2:**
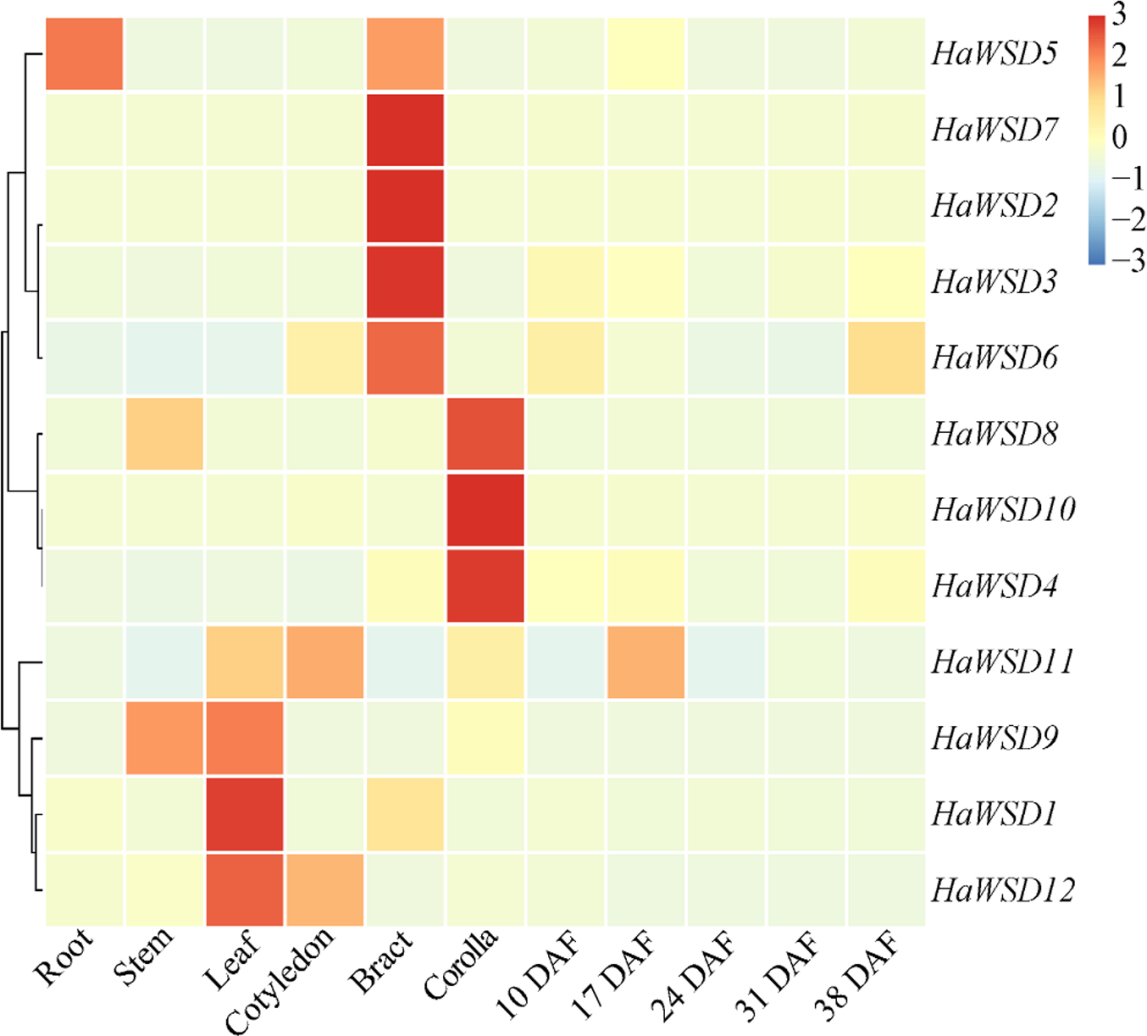
The expression pattern of *HaWSD* genes in different sunflower tissues and developing seeds from 10 to 38 DAF (days after flowering) stages by qRT-PCR. Sunflower endogenous 18S rRNA gene (AF1057577) was used as reference gene and root samples were used for reference tissues. The transcript levels are shown with graded color scale from sky-blue to red.

The expression levels of *HaWSD* genes in six tissues of sunflower including roots, stems, leaves, cotyledon, bracts, corollas, and developing seeds (10, 17, 24, 31 and 38 DAF) were examined by qRT-PCR (**Figure 2**). *HaWSD* genes exhibited high expression in leaves, bracts or corollas, but low expressed during seed development. Interestingly, *HaWSD5* was preferentially expressed in roots. *HaWSD1*, *HaWSD9* and *HaWSD12* were highly expressed in leaves, while *HaWSD8* and *HaWSD9* were highly expressed in stems. These analyses showed that *HaWSD* genes may be involved in wax accumulation of different tissues.

### Expression profiles of *HaWSD* genes under abiotic stresses

**Figure 3:**
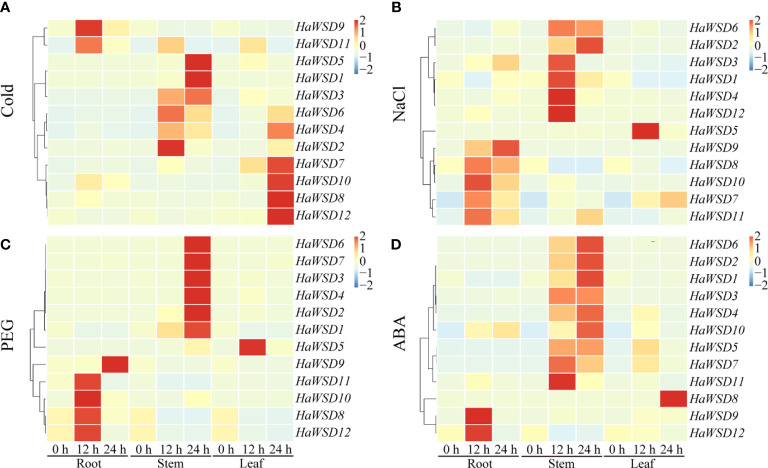
The expression pattern of *HaWSD* genes in sunflower under different abiotic stresses. Four-week-old sunflower seedlings were treated with stress conditions, including cold (4°C) **(A)**, NaCl (200 mM) **(B)**, drought (15% PEG 6000) **(C)**, and the signaling hormone ABA **(D)** at the indicated time points (0, 12, and 24 h) in roots, stems and leaves, respectively. The qRT-PCR was used to study the transcript levels of *HaWSD* genes under abiotic stresses. The transcript levels are shown with graded color scale from sky-blue to red.

To evaluate the function of *HaWSD* genes in response to abiotic stresses, the transcript levels of 12 *HaWSD* genes in sunflower stems, roots and leaves under drought, cold (4°C), ABA and salt (NaCl) stresses were analyzed by qRT-PCR. The results revealed that the expression pattern of *HaWSD* genes varied under abiotic stresses, and almost all the *HaWSD* genes were induced in sunflower roots, leaves or stems **(** **Figure 3** **)**.

Under cold (4°C) stress, two genes (*HaWSD*9, and -*11*) experienced significant up-regulation in roots at 24 h and then became decreased, six (*HaWSD1-6*) in stems at 12 and 24 h, and five genes (*HaWSD4*, *-7*, *-8*, *-10*, and -*12*) were induced in leaves at 24 h (**Figure 3A**). Under 200 mM NaCl stress, five genes (*HaWSD7-11*) were significantly up-regulated in roots, six (*HaWSD1-4, -6*, and *-12*) in stems at 12 and 24 h, and only one gene (*HaWSD5*) was up-regulated in leaves at 12 h (**Figure 3B**). Under 15% PEG-simulated drought stress, four genes (*HaWSD8, -10, -11*, and *-12*) peaked at 12 h and *HaWSD9* peaked at 24 h in roots, six genes (*HaWSD1-4, -6*, and *-7*) significantly induced in stems at 24 h, while only one gene (*HaWSD*5) was induced in leaves at 12 h (**Figure 3C**). These findings suggest that *HaWSD* genes are involved in the response to drought stress through regulation of wax esters accumulation (Patwari et al., 2019). Under 100 μM ABA treatment, a total of 9 *HaWSD* genes were significantly up regulated in stems at 12 and 24 h, two (*HaWSD9* and *-12*) in roots at 12 h, and only one gene (*HaWSD8*) was remarkably enhanced in leaves at 24 h (**Figure 3D**). Overall, *HaWSD* genes were mainly induced in both roots and stems of sunflower under PEG and NaCl treatments; most were induced in stems and leaves under cold treatment; However, *HaWSD* genes were mainly induced in stems in response to ABA. These results indicated that *HaWSD* genes may play important roles in sunflower growth and stress responses.

### Subcellular localization of *HaWSD9*

**Figure 4:**
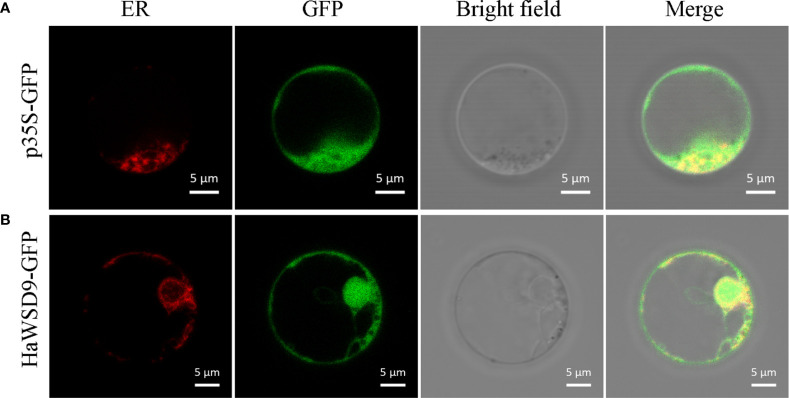
Subcellular localization of HaWSD9 in Arabidopsis protoplast cell. Endoplasmic reticulum (ER) marker fusion (HDEL-RFP) was used as an ER marker. Scale bar = 5 μm. **(A)** The empty p35S-GFP vector was used as a control. **(B)** Co-localization of HaWSD9-GFP (green) with ER marker (red).

In order to investigate the subcellular localization of HaWSD9, the HaWSD9-GFP were co-expressed with the ER marker (HDEL-RFP) in Arabidopsis protoplasts. Co-localization of HaWSD9 and the ER marker was observed, as expected (**Figure 4A**). In contrast, the green signal of HaWSD9 (**Figure 4B**) was completely co-localized with HDEL-RFP, indicating that HaWSD9 was located in the ER, which was similar to Arabidopsis AtWSD1 (Li et al., 2008).

### Heterologous expression of *HaWSD9* in yeast TAG deficient mutant

**Figure 5:**
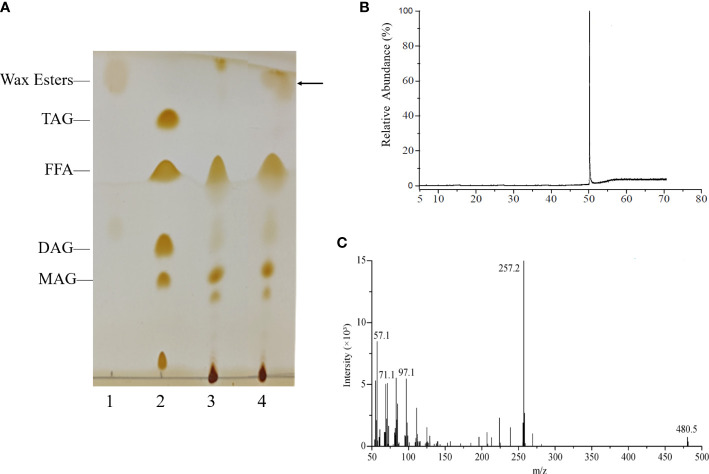
Wax ester production by recombinant yeast H1246 expressing *HaWSD9*. **(A)** Thin-layer chromatography (TLC) of the neutral lipids synthesized in recombinant yeasts mutant strain H1246. Lane 1, Wax ester standard (Palmityl palmitate); Lane 2, TAG standard; Lane 3, TAG-deficient quadruple mutant strain H1246 with empty pYES2 vector, the negative control, fed with 0.1% (w/v) palmitic acid and 0.1% (w/v) hexadecanol (C16 alcohol); Lane 4, the pYES2-*HaWSD9*-overexpressing yeast H1246 fed with 0.1% (w/v) palmitic acid and 0.1% (w/v) hexadecanol. **(B, C)** GC-MS analysis of wax esters in *HaWSD9*-overexpressing yeast H1246 transformants. **(B)**, Wax esters produced by transgenic yeast were separated from TLC plate and analyzed by GC-MS. **(C)** Mass spectra of hexadecyl (C16) palmitate (molecular ion m/z=257.2, corresponding to C32 wax ester).

To verify whether enzyme from the *HaWSD9* gene has TAG synthesis or wax ester activities, it was heterologous expressed in *S. cerevisiae* mutant strain H1246 (Sandager et al., 2002). The H1246 transformed with empty plasmid pYES2 was treated as the negative control. The yeast H1246 cells expression of *HaWSD9* gene were grown in galactose-supplemented media and in the presence of palmitic acid (C16:0) and hexadecanol (C16 alcohol). Lipids were then extracted from the recombinant strain and analyzed by TLC analysis. As shown in the TLC profile (**Figure 5A**), *HaWSD9-*introduced yeasts exhibited the remarkable wax esters accumulation compared with the empty vector control, whereas there were no detectable amounts of TAG formed in the recombinant line under the medium conditions with palmitic acid and hexadecanol. The exact structures of these wax esters were purified *via* TLC and determined by GC-MS assay as ester hexadecyl palmitate (C32) (**Figures 5B, C**). These results suggested that HaWSD9 was enzymatically toward wax ester synthesis and can use C16 fatty acid and C16 alcohol as substrates but can’t use DAG as substrate *in vivo*.

### Phenotypic analysis of Arabidopsis mutants overexpressing *HaWSD9*

**Figure 6:**
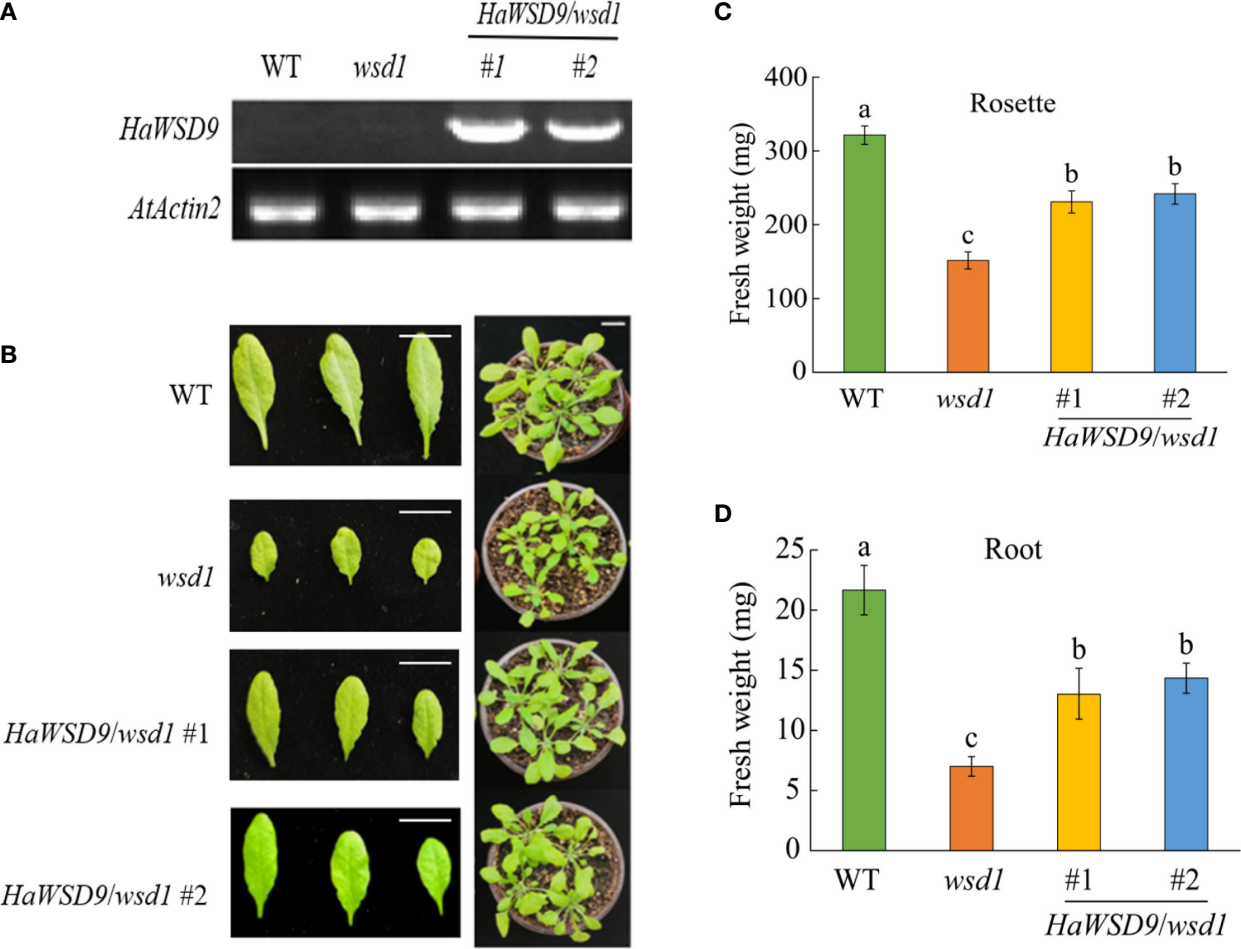
Phenotypes of *HaWSD9* transgenic Arabidopsis, *wsd1* and WT. *HaWSD9* was overexpressed in Arabidopsis *wsd1* mutant under the control of the CaMV 35S promoter. **(A)** Expression of *HaWSD9* in leaves of two *HaWSD9* overexpression lines (*HaWSD9*/*wsd1*#1 and *HaWSD9*/*wsd1*#2), *wsd1* and wild type Arabidopsis. Total RNA was isolated from two transgenic lines, *wsd1* and WT, and semi-quantitative RT-PCR was used for expression analysis. WT: wild-type Arabidopsis (Col-0); *wsd1*: Arabidopsis mutant *wsd1* line; #1 and #2: the two lines of *HaWSD9* overexpressing in *wsd1* mutant. AtActin2 (At3g18780) was used as the control. **(B)** Phenotype of the WT, *wsd1* and *HaWSD9*-overexpressing *wsd1* transgenic lines of 4-week-old growth. **(C)** Statistics of fresh weight on Roots in WT, *wsd1* and transgenic lines. **(D)** Statistics of fresh weight on rosette leaves in WT, *wsd1* and transgenic lines. Different lowercase letters indicate significant differences at p < 0.05.

The cDNA of *HaWSD9* was cloned behind the CaMV35S promoter and transformed into Arabidopsis *wsd1* mutant plants. Transcription levels of *HaWSD9* in transgenic lines were detected by semi-quantitative RT-PCR. Two lines, *HaWSD9/wsd1#1* and *HaWSD9/wsd1#2*, with strong *HaWSD9* expression levels were selected for further analysis (**Figure 6A**). Next, to evaluate the effect of *HaWSD9* gene expression on the growth of Arabidopsis, we measured the fresh weight of rosette leaves and roots of WT, *wsd1* and two *HaWSD9* overexpressing lines after 4 weeks of growth (**Figure 6B**). The overexpression lines showed higher fresh weight in roots and rosette leaves compared with *wsd1* (**Figures 6C, D**).

### Epicuticular wax quantity and composition is altered in *HaWSD9* overexpression lines

**Figure 7:**
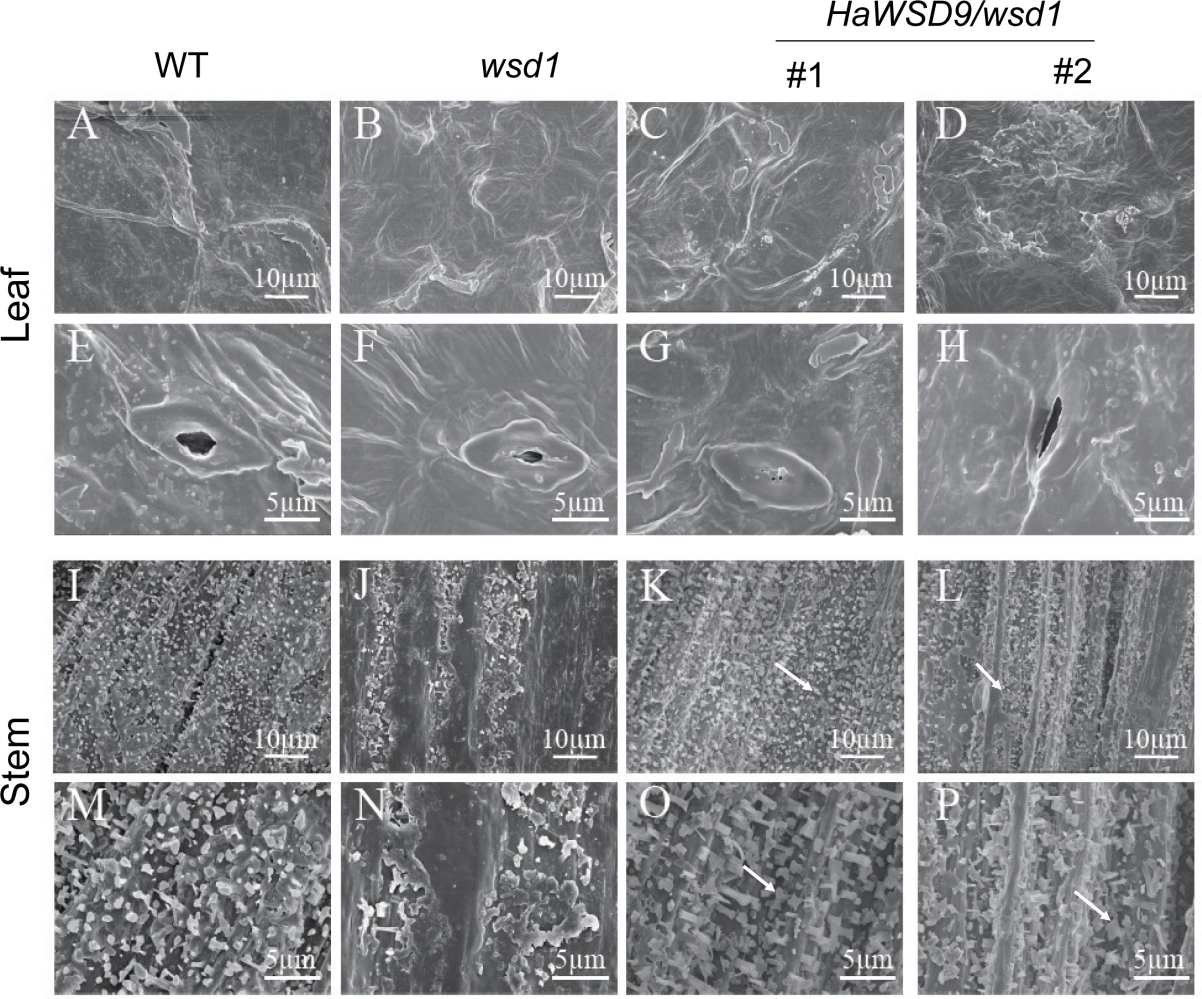
Scanning electron microscopy of the epicuticular wax of the leaf and stem surface of WT, *wsd1* and *HaWSD9* transgenic lines in *wsd1*. The stem surface of *HaWSD9* overexpressing lines (#1 and #2) were covered with much more wax crystals and wax deposition than *wsd1* mutant (arrows point to the epicuticular wax crystals in transgenic lines). **(A–H)** leaf; **(I–P)**: stem; Bars, 5 μm in **(E–H, M–P)**, 10 μm in **(A–D, I-L)**.

To further elucidate whether the overexpression of *HaWSD9* is associated with wax biosynthesis, scanning electron microscopy (SEM) was performed to investigate the cuticular waxes deposited on stem and leaf surfaces in Arabidopsis. Much more epicuticular wax crystals were observed on the stems of *HaWSD9* transgenic lines compared with *wsd1* mutant (**Figure 7**).

**Figure 8:**
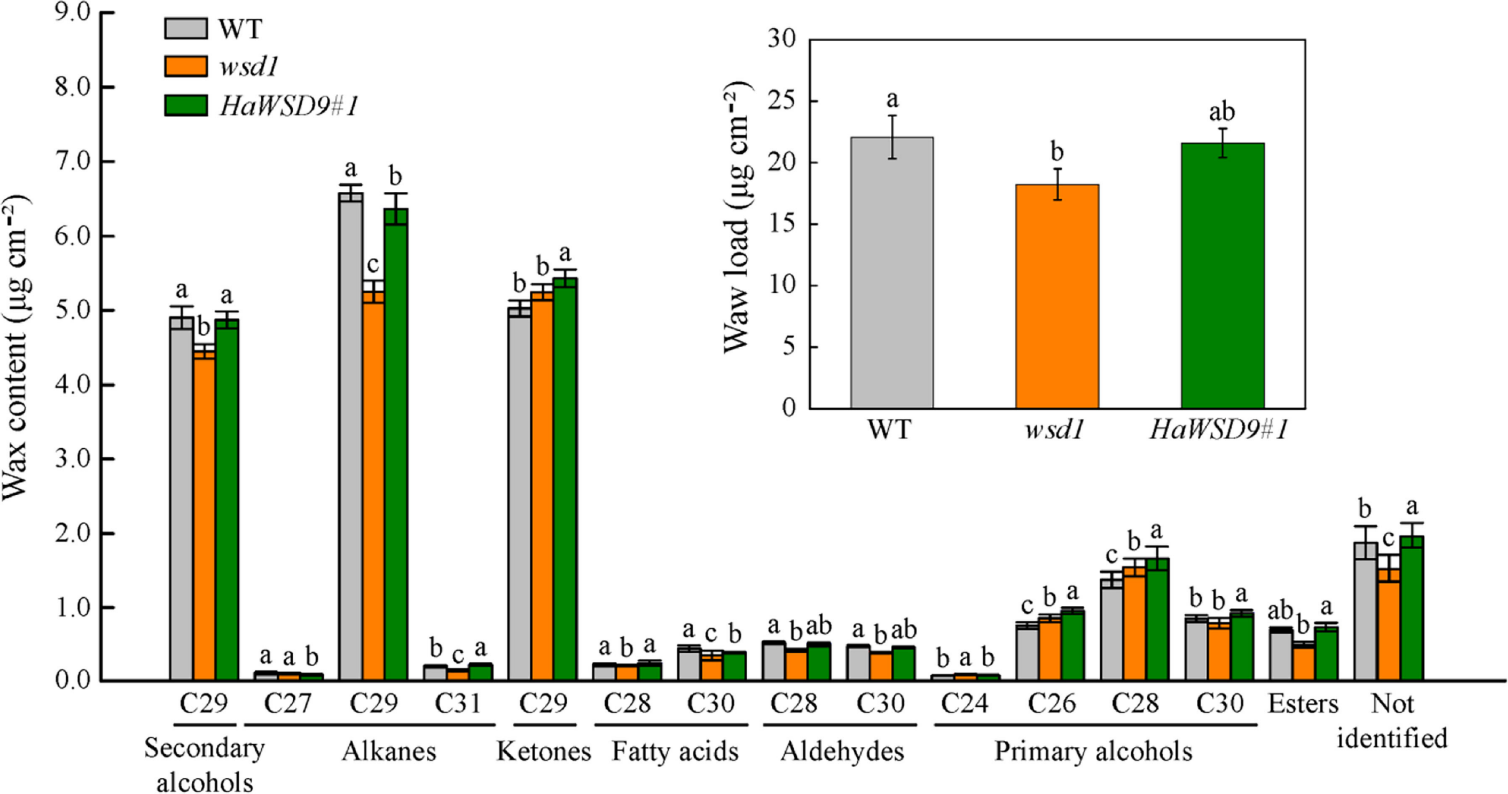
Cuticular wax load and composition in stems of WT, *wsd1* and *HaWSD9* overexpressing plants. Stems of 6-week-old WT, *wsd1* and *HaWSD9* transgenic plants were used for cuticular wax loads analysis. Data represents mean ± SD of three independent biological replicates. Lowercase letters indicate statistically significant difference (p < 0.05).

To examine whether *HaWSD9* affects wax composition, we examined cuticular wax content and composition in stem of *HaWSD9-*overexpressing lines by GC-MS (**Figure 8**). The total wax loads in *wsd1* stem were lower than that in WT stem (22.7% decrease), while the *HaWSD9-* overexpressing lines showed a 18.4% increase in total wax loads as compared to *wsd1*. Therefore, overexpression of *HaWSD9* in *wsd1* resulted in a higher wax ester accumulation on the stem surface. Additionally, the composition of cuticular wax in Arabidopsis stem was altered significantly (**Figure 8**). Stem primary (C26 and C28) and secondary alcohols (C29), alkanes (C29 and C31), fatty acids (C28 and C30), and total wax esters were increased significantly in *HaWSD9* overexpressing lines compared to *wsd1*.

### *HaWSD9* altered the cuticular permeability in Arabidopsis

**Figure 9:**
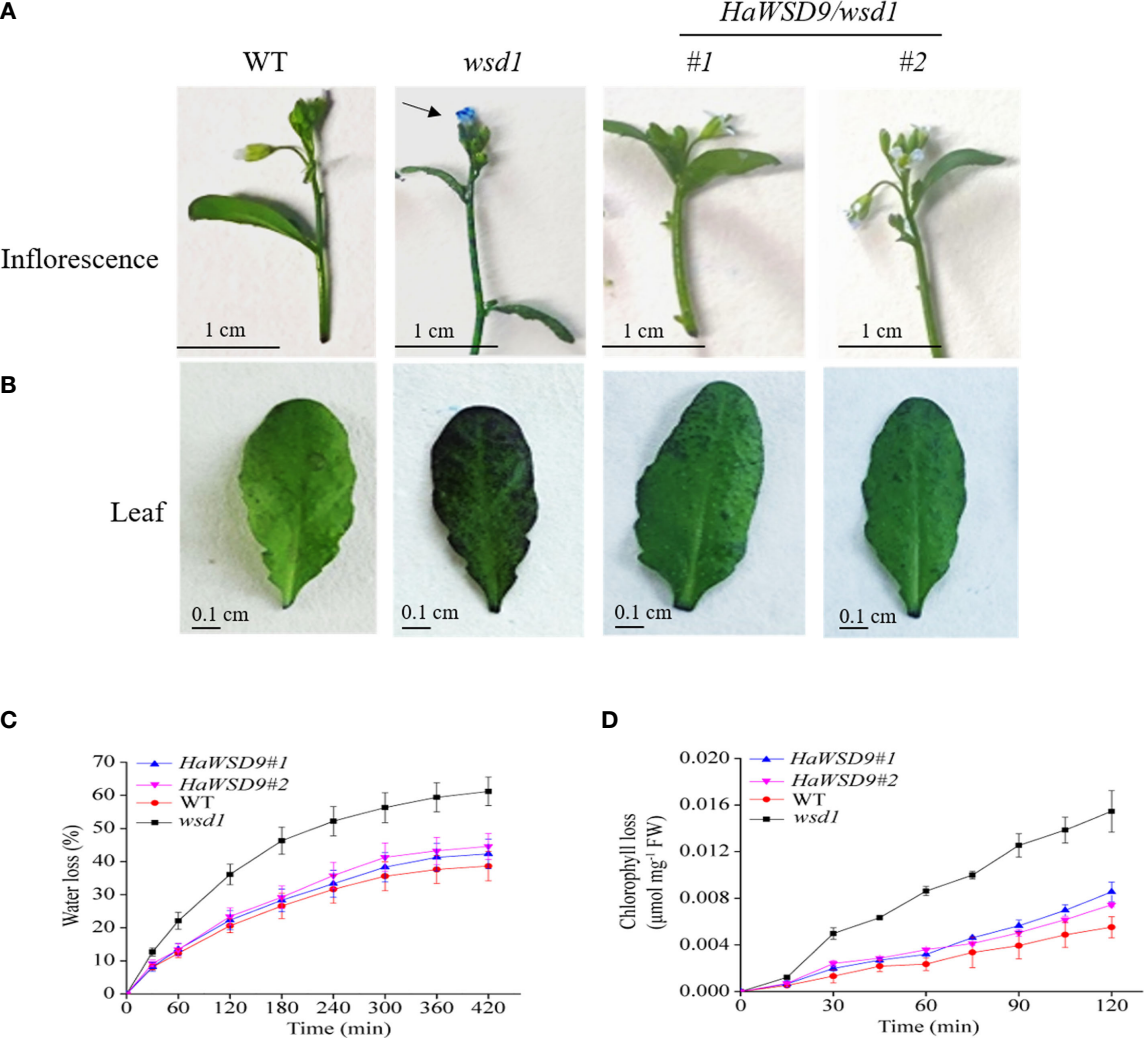
Alterations in cuticle permeability of *HaWSD9* overexpressing in *wsd1* transgenic plants. **(A, B)** Toluidine blue (TB) staining of inflorescences **(A)**, and leaves **(B)** of WT, *wsd1* and *HaWSD9* overexpressing lines. **(C)** Water loss rate in leaves of WT, *wsd1* and *HaWSD9* overexpressing lines. **(D)** Chlorophyll leaching assay in leaves from WT, *wsd1* and *HaWSD9* overexpressing lines. FW, fresh weight.

In plants, the permeability of epidermis is usually related to the content of epidermal wax. In general, Arabidopsis wax-deficient mutants are more water permeable than the wild-type, most probably due to the increase of water permeability in leaves (Samuels et al., 2008). Toluidine blue (TB) staining is a good method to detect the epidermal permeability of plant tissues. Plant leaf defect types are more likely to be stained with TB (Yang et al., 2021). To determine whether the *HaWSD9* transgenic lines (#1 and #2) is related to changes in epidermal permeability, TB staining was performed in leaves and inflorescences of WT, *wsd1* and *HaWSD9* overexpressing lines (**Figures 9A–D**). The results showed that the leaves and inflorescences of the *HaWSD9* overexpressing lines were less stained than those of *wsd1* (**Figures 9A, B**). The leaf water loss rate in *HaWSD9* overexpressing lines was similar with WT, but was faster in *wsd1* mutants (**Figure 9C**). Furthermore, the chlorophyll leaching rate was measured in rosette leaves from *HaWSD9* overexpressing lines (#1 and #2), *wsd1* mutant and WT. The rosette leaves of *HaWSD9* overexpressing lines showed reduced chlorophyll leaching compared to *wsd1* leaves (**Figure 9D**). These results indicated that *HaWSD9* is closely related to the leaf cuticle permeability, chlorophyll leaching and water loss, and it might play an important role in cuticular wax biosynthesis and drought tolerance.

### *HaWSD9* increased Arabidopsis abiotic stress tolerance in MS media

**Figure 10:**
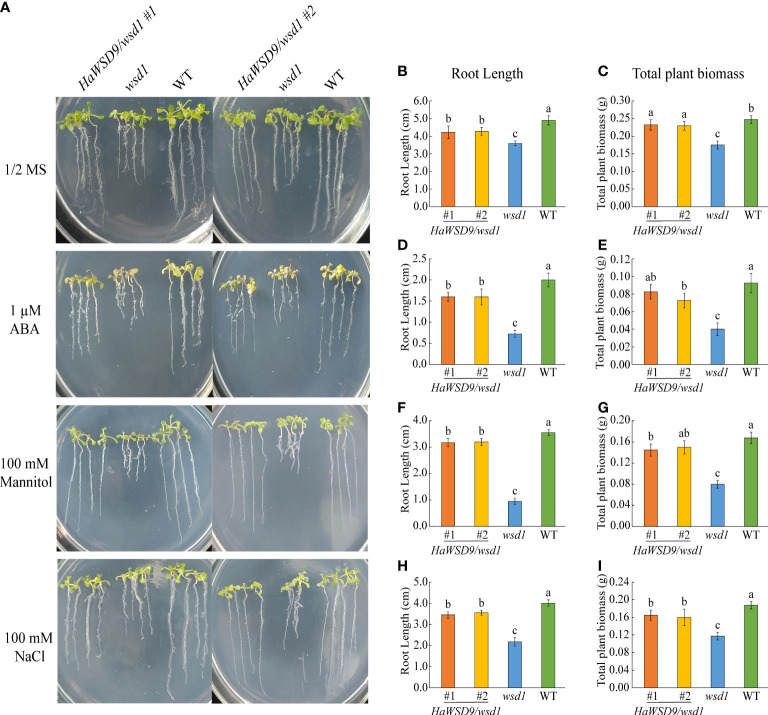
Screening *HaWSD9* overexpressing transgenic plants for tolerance to ABA, mannitol, and salt stresses. Wild type (Col-0, WT), *wsd1* and *HaWSD9* transgenic lines were germinated on 1/2 MS medium alone or supplemented with mannitol (100 mM), NaCl (75 mM), and ABA (1 μM) for 21 days. **(A)** Photographs of WT, *wsd1* and *HaWSD9* transgenic (#1 and #2) seedlings. **(B, D, F, H)** Root length (cm) measurements under control, ABA, mannitol, and NaCl treatments, respectively. **(C, E, G, I)** Plant total biomass (g) under control, ABA, mannitol, and NaCl treatments, respectively. Data represents mean ± SD of three independent biological replicates. Lowercase letters indicate statistically significant difference (p < 0.05).

To investigate whether the accumulation of cuticular wax enhanced *HaWSD9* transgenic Arabidopsis in resistance to abiotic stress, seeds of Arabidopsis WT, *wsd1* mutant and two *HaWSD9* transgenic lines were grown on 1/2 MS medium with 75 mM NaCl, 100 mM mannitol or 1 μM ABA. After three weeks, the root length and total plant biomass of all the treatments were measured. Compared with *wsd1* mutant seedlings, transgenic seedlings exhibited a increase in the root length and plant biomass under normal conditions (**Figure 10A**). Quantitative analysis revealed an increase of the total root length by 18% and of the plant biomass by 34% in transgenic plants compared with *wsd1* (**Figures 10B, C**). When grown on 1/2 MS medium with ABA, NaCl, and osmotic stress conditions, the total root biomass and root length were statistically increased in two transgenic lines compared with *wsd1* mutant (**Figures 10D–I**). The transgenic lines showed a 59% to 230% increase in total root length and a 36% to 106% increase in total biomass compared with the *wsd1* mutant under abiotic stress conditions. These results suggested *HaWSD9* was involved in physiological responses to salt, drought and ABA-mediated stress responses.

### Expression of *HaWSD9* enhances drought stress in transgenic Arabidopsis under greenhouse conditions

**Figure 11:**
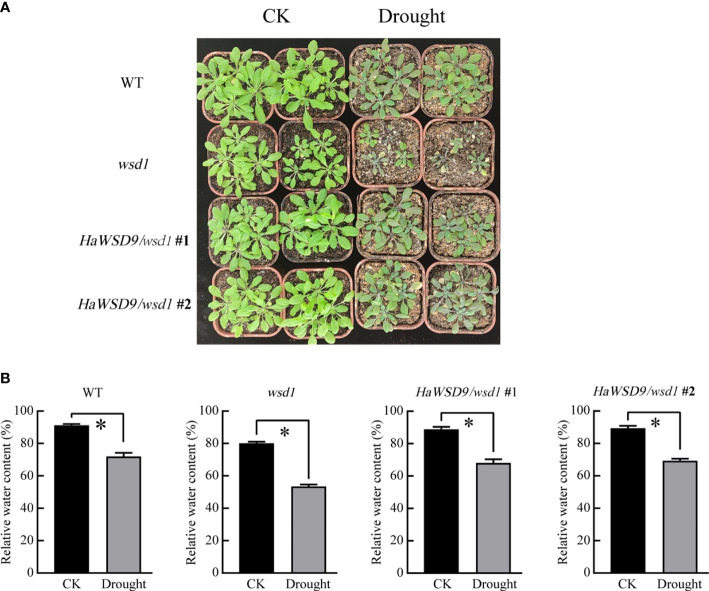
*HaWSD9* overexpression confers drought tolerance. **(A)** 4-week-old plant phenotypes of WT, *wsd1* and *HaWSD9* transgenic plants under normal conditions (CK) and after water withholding for 10 days. **(B)** RWC content of the detached leaves. Error bars show the standard deviations for three independent replicates. Asterisks indicate statistically significant differences compared to CK (*P < 0.05).

To further investigate the function of *HaWSD9* in the drought stress response in soil, 4-week-old *HaWSD9* transgenic plants as well as WT and *wsd1* mutant were subjected to drought stress by withholding water for 10 d. The results showed that about 10 days of stopping watering, the *HaWSD9* transgenic plants exhibited a strong drought-tolerant phenotype compared to *wsd1* mutant (**Figure 11A**). The leaves in *wsd1* mutant were much smaller and exhibited more serious leaf wilting than WT and transgenic plants. Then we measured the relative water content (RWC), and found the content of RWC was decreased by 26.7% in *wsd1* (**Figure 11B**), while only 20% reductions of RWC in *HaWSD9* transgenic plants, which similar with that of WT.

## Discussion

In plants, wax ester synthase/acyl-CoA: diacylglycerol acyltransferase is a crucial enzyme involved in wax ester synthesis. WSD belong to membrane bound DGAT family, which form at least four phylogenetically different groups. Among them, the WSD group is the least explored in plant, and analysis of *WSD* gene family members in sunflower has not been reported so far. In the present study, we performed a comprehensive analysis of the *HaWSD* gene family and determined their function in wax ester biosynthesis and response to abiotic stresses.

Based on the bioinformatic analysis, 12 *HaWSD* genes were identified from sunflower genome, which were similar to Arabidopsis (11). HaWSDs have diverse sequence (25.2%–88.2% amino acid sequence identity) and size, range from 455 to 666 resides in length. In addition, like the Arabidopsis AtWSD1, all HaWSD proteins contain a highly conserved active-site motif (HHXXXDG) in the N-terminal domain (**Figure 1B**), which was essential for acyl-CoA acyltransferase activity and responsible for the ester bond (Kalscheuer and Steinbüchel, 2003). Whether these proteins have the activity in wax ester or DGAT biosynthesis needs to be further investigated.

Previous study indicated that many of the Arabidopsis *AtWSD* genes were higher expressed in flowers and siliques and play an important role in wax accumulation in these tissues (Patwari et al., 2019). Among them, *AtWSD1* was expressed highest in the stem epidermis and play a key role in the biosynthesis of wax esters in Arabidopsis stem (Li et al., 2008). Gene expression analysis showed that most of *HaWSD* genes were expressed in sunflower stems, leaves, cotyledons, bracts or corollas (**Figure 2**), but exhibited low expression levels in sunflower developing seeds, suggesting that *HaWSD* genes display weak function as TAG biosynthesis.

To further analysis the functions of *HaWSD* genes, *HaWSD9* gene, with highly expressed in sunflower leaves and stems, was selected and heterologous expressed in yeast mutant H1246. Induction of *HaWSD9* in yeast H1246 restored wax ester biosynthesis after feeding of palmitic acid (C16:0) and hexadecanol (C16 alcohol) but failed to restore TAG biosynthesis (**Figure 5A**), indicating that *HaWSD9* does not exhibit DGAT activity *in vivo*. Similar findings have also been reported previously in AtWSD1 from Arabidopsis (Li et al., 2008) and PhWS1 from petunia (King et al., 2007). Furthermore, overexpression of *HaWSD9* in Arabidopsis mutant *wsd1* increased the deposition of epicuticular waxes and altered wax composition on the stems compared to the *wsd1* mutant (**Figure 8**). These indicated that *HaWSD9* may be involved in the wax ester biosynthesis of sunflower.

WS/DGAT also play important roles in plant resistance to abiotic stresses in addition to cuticular wax synthesis (Abdullah et al., 2021). Arabidopsis *AtWSD1* was up regulated under several abiotic stresses and activate wax biosynthesis in response to environmental stresses. Sunflower growth and productivity are greatly affected by abiotic stresses such as salinity, drought and cold (Allinne et al., 2009; Fulda et al., 2011; Szymańska et al., 2022). The expression of 12 *HaWSD* genes under different stresses revealed that most of genes can be induced significantly by exogenous ABA and PEG-induced drought stress in sunflower stems, leaves and roots (**Figure 3**). Drought stress causes osmotic stress in plants, which further causes dehydration in plant cells. Plants have evolved complex signaling pathways to resist osmotic stress, including ABA-dependent and ABA-independent pathways (Yoshida et al., 2014). These results suggested that *HaWSD* genes involve in regulating drought tolerance through ABA-dependent signaling pathways by changing cuticular wax composition. The up regulation of *HaWSD* genes transcripts also observed under cold, and NaCl stresses, suggesting that *HaWSD* genes may play important regulatory roles in response to a variety of stresses.

Overexpression of *HaWSD9* in Arabidopsis *wsd1* mutant showed better resistance phenotypes than *wsd1* in response to exogenous ABA, mannitol, and NaCl when grown on 1/2 MS media (**Figure 10**). Moreover, *HaWSD9* overexpressing plants showed a higher tolerance phenotype than *wsd1* when plants growing on soil were exposed to drought (**Figure 11**). It has been demonstrated in plants that there is a relationship between wax content and epidermal permeability (Lü et al., 2009). Epidermal permeability is often measured by TB staining and chlorophyll extraction rate (Wang et al., 2014). In a bloomless mutant (epicuticular wax mutant) of sorghum, which possessed 15% lower amount of wax on the epidermal surface, have been reported to have increased epidermal permeability and nine-time water loss (Burow et al., 2008). In apple, ectopic expression of *MdCER2* in Arabidopsis increased epidermal wax accumulation, was positively correlated with the reduced chlorophyll leaking and water loss (Zhong et al., 2020). Here, TB staining, leaf water loss and chlorophyll leaching assay was carried out to investigate the function of *HaWSD9* in Arabidopsis permeability. We found that *wsd1* mutant is more easily stained than that of *HaWSD9* overexpressing Arabidopsis lines and WT (**Figure 9**). In addition, the *HaWSD9* overexpressing lines reduced the chlorophyll leaking rates and water loss in leaves compared with *wsd1*. Therefore, we speculate that the reduction in chlorophyll leaking, and water loss was associated with reduced cuticle permeability. Furthermore, GC analysis results indicated that overexpressing *HaWSD9* increased the total wax load in Arabidopsis stem compared with *wsd1* mutant plants, which mainly resulted the increase in primary (C26 and C28) and secondary alcohols (C29), alkanes (C29 and C31), fatty acids (C28 and C30), and total wax esters (**Figure 8**). It has been reported that, under water deficit stress, transgenic Arabidopsis overexpressing *AtWSD* increased 40-70% of the cuticle waxes, and the major compounds were found to be long chains n-alkanes (C29 and C31) (Abdullah et al., 2021). Therefore, it is reasonable to believe that *HaWSD9* is involved in regulating the cuticular wax biosynthesis and play an important role in drought resistance.

## Conclusion

In this study, genome-wide analysis of sunflower genome identified 12 *HaWSD* genes. We analyzed their similar conserved motif, chromosomal location, and phylogenetic relationship with Arabidopsis. *HaWSD* genes were differentially expressed in various tissues, during sunflower seed development, and were induced by drought, high salt, cold and ABA. Ectopic expression in yeast mutant demonstrated that *HaWSD9* exhibits wax ester synthase activity but no DGAT activity *in vivo*. Overexpression of *HaWSD9* in Arabidopsis *wsd1* mutant increased the accumulation of wax content, and significantly enhanced plant drought resistance. This study will be useful for improving drought stress resistance in sunflower.

## Data availability statement

The datasets presented in this study can be found in online repositories. The names of the repository/repositories and accession number(s) can be found in the article/**Supplementary Material**.

## Author contributions

LS conceived and designed the experiments. CZ and JY performed the experiments. WM and LZ analyzed the data. CZ wrote the manuscript. LS and CZ revised the manuscript. All authors contributed to the article and approved the submitted version.

## Funding

This work was supported by the National Natural Science Foundation of China (31760064 and 31360052).

## Acknowledgments

We thank Prof. Zhifan Yang (College of Life Sciences, Hubei University, China) for providing the H1246 yeast cells.

## Conflict of interest

The authors declare that the research was conducted in the absence of any commercial or financial relationships that could be construed as a potential conflict of interest.

## Publisher’s note

All claims expressed in this article are solely those of the authors and do not necessarily represent those of their affiliated organizations, or those of the publisher, the editors and the reviewers. Any product that may be evaluated in this article, or claim that may be made by its manufacturer, is not guaranteed or endorsed by the publisher.
