# Supplementary material for: Genome-wide analysis of the WSD family in sunflower and functional identification of HaWSD9 involvement in wax ester biosynthesis and osmotic stress
Source: Front Plant Sci. 2022 Sep 23;13:975853. doi: 10.3389/fpls.2022.975853 (PMC9539440; doi:10.3389/fpls.2022.975853)
Supplement: Supplementary file 1 [file Table_1.doc]

**Supplementary Table S1. Primer sequences used for qRT-PCR*, HaWSD9* cloning, vector construction, and transgenic confirmation.**

|  | | **Primer use** | **Primer Name** | | **Sequences (5'-3')** | |
| --- | --- | --- | --- | --- | --- | --- |
|  |  |  |  |  | **Forward** | **Reverse** |
|  | qRT-PCR | | | q-HaWSD1 | GAGGATACCGAAACGCCGAT | ACCTTCGTCTTTTTGCCCCT |
|  |  | | | q-HaWSD2 | TGGATGCCGCTAAACACGTC | CGGCGATGAAACCGTGAGAT |
|  |  | | | q-HaWSD3 | TGGAGGATAGGCCAAGACGA | TTGTTTCCCCATGTGCCATCT |
|  |  | | | q-HaWSD4 | GAAAGGAGAGGACCACGACC | GCTCCCGTTTTCCTTGTCCA |
|  |  | | | q-HaWSD5 | GTTGGTCACGGACAAACACG | ACGATAGAAGCGAAACGCCA |
|  |  | | | q-HaWSD6 | GGGGCCAAAGATGACCAAGA | CCACCACCGTTGCTTCTAGT |
|  |  | | | q-HaWSD7 | TTCGTTAGGCGACGGTTTGT | GCCCATGCTGCCTTTTAACG |
|  |  | | | q-HaWSD8 | TGTGTGCACTGGGCTGTTTC | ACACCCGCGATTTCACTGTATC |
|  |  | | | q-HaWSD9 | CACTAAAAGGGTCCGCAGGT | GTCGAACGCCTTTGGGAATG |
|  |  | | | q-HaWSD10 | GCACGACGAACCACTATCCC | TCCGCCAATGTTCATGTCCG |
|  |  | | | q-HaWSD11 | AGCGTGTTTGGCCTCGAAC | GTGAGGTCGGCTAGGTATTCG |
|  |  | | | q-HaWSD12 | TGTGTGGAGGATGTGACCATGT | GGTCTTCCCCCTTCACTTCAAC |
|  |  | | | Ha18s rRNA | CTACCACATCCAAGGAAGGCAG | CGACAGAAGGGACGAGTAAACC |
|  | *HaWSD9* cloning | | | P1 | ATGAAACAAAGTATATCTCT | TTACTTGTCTTTGGTACTCT |
|  | pAN580-*HaWSD9* construction | | | P2 | TCCCCCGGGATGGGTTCCTCAGAAGGTCT  (*Sma*I site is underlined) | CGCGGATCCCTTGTCTTTGGTACTCTCTT  (*Bam*HI site is underlined) |
|  | PYES2-*HaWSD9* construction | | | P3 | CGCAAGCTTATGAAACAAAGTATATCTCT  (*Hind*Ⅲ site is underlined) | TGCTCTAGATTACTTGTCTTTGGTACTCT  (*Xba*I site is underlined) |
|  |  | | |  |  |  |
|  | pCAMBIA2300- *HaWSD9* construction | | | P4 | TCCCCCGGGATGGGTTCCTCAGAAGGTCT  (*Sma*I site is underlined) | CGCGGATCCTTACTTGTCTTTGGTACTCT  (*BamHI* site is underlined) |
|  | Arabidopsis *wsd1* mutant identification | | | LP | TTGAATTTAATAGCCAGGGCC |  |
|  |  | | | BP | ATTTTGCCGATTTCGGAAC |  |
|  |  | | | RP |  | TATTGGCGTTGTCTCGATCTC |
|  | *HaWSD9* expression in transgenic lines | | | P5 | CACTAAAAGGGTCCGCAGGT | GTCGAACGCCTTTGGGAATG |
|  | *AtActin2* (At3g18780) | | | P6 | GTTGGTGATGAAGCACAATCCAAG | CTGGAACAAGACTTCTGGGCATCT |
