# Supplementary figures and images for: Genome-wide analysis of the WSD family in sunflower and functional identification of HaWSD9 involvement in wax ester biosynthesis and osmotic stress
Source: Front Plant Sci. 2022 Sep 23;13:975853. doi: 10.3389/fpls.2022.975853 (PMC9539440; doi:10.3389/fpls.2022.975853)

## Slide 1
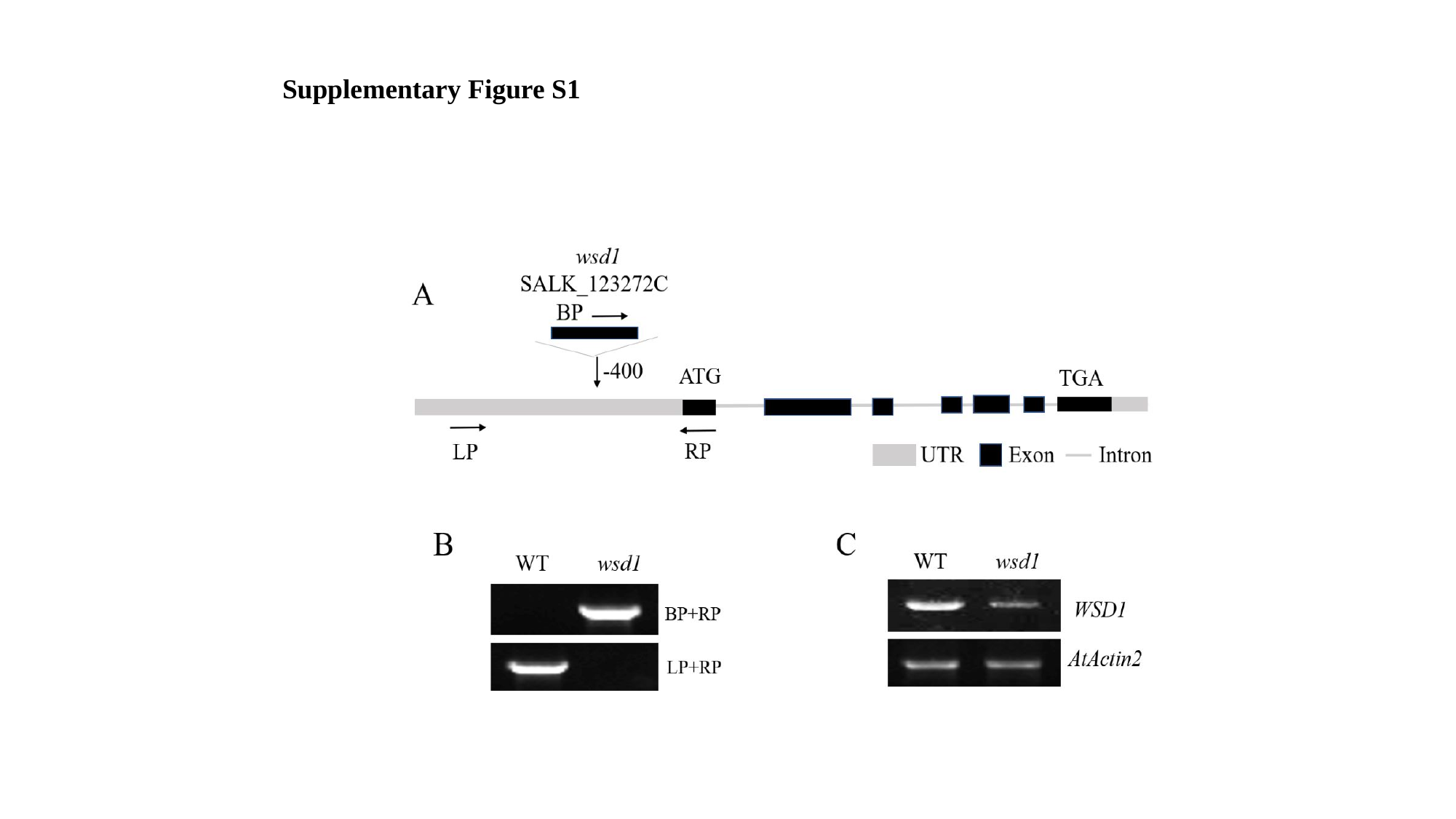

Supplementary Figure S1

## Slide 2
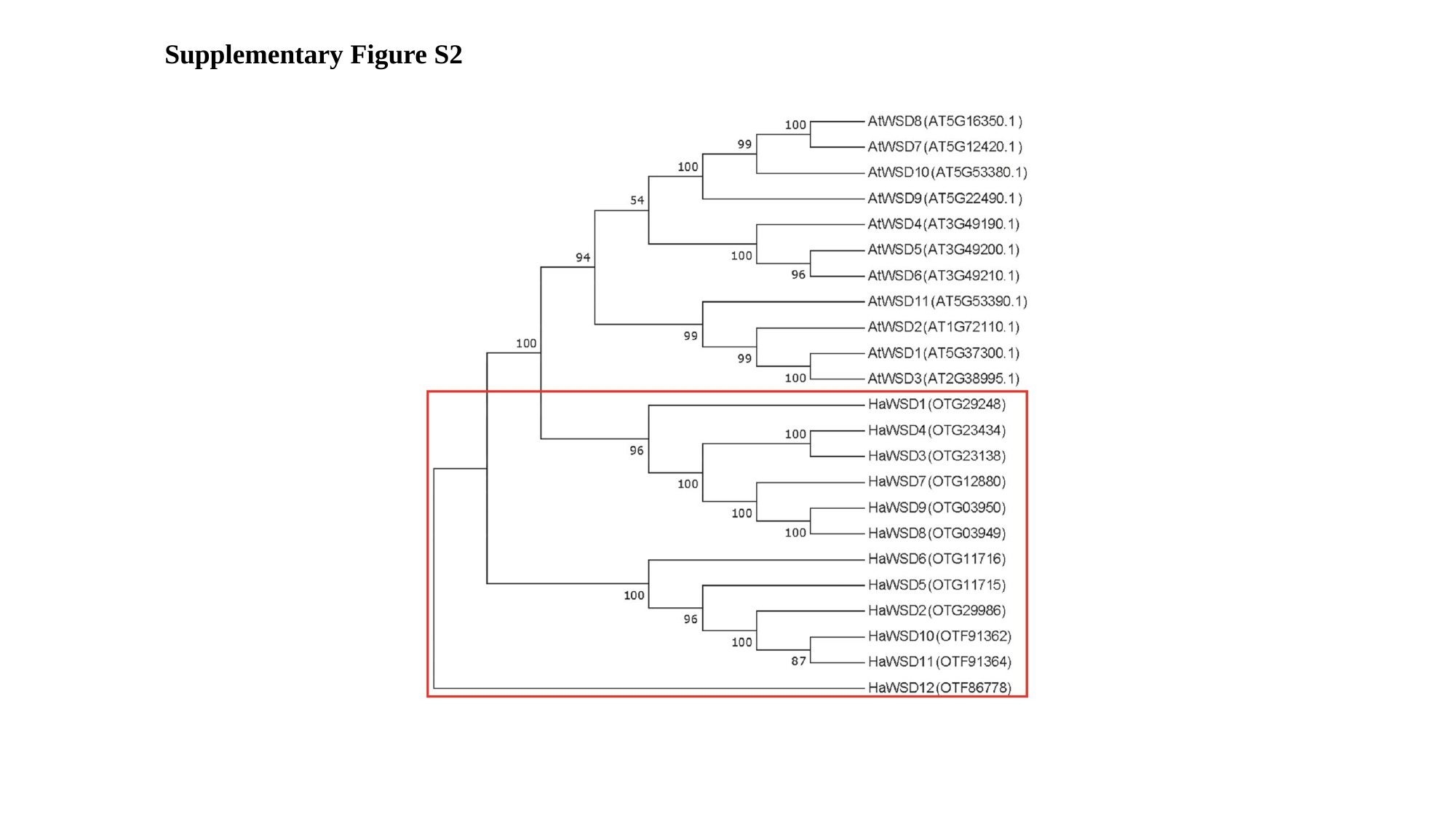

Supplementary Figure S2

Supplement: Supplementary file 3 [file Presentation_1.pptx]
