# Supplementary material for: Genome-wide analysis of the WSD family in sunflower and functional identification of HaWSD9 involvement in wax ester biosynthesis and osmotic stress
Source: Front Plant Sci. 2022 Sep 23;13:975853. doi: 10.3389/fpls.2022.975853 (PMC9539440; doi:10.3389/fpls.2022.975853)
Supplement: Supplementary file 4 [file DataSheet_1.docx]

**Supplementary Figure S1**|Identification of the *wsd1* mutant in Arabidopsis

1. Schematic diagram of Arabidopsis *WSD1* gene structure with the T-DNA insertion mutant. Arrow indicate T-DNA insertion sites. **(B)** PCR analysis of Arabidopsis *wsd1* mutant. Homozygous mutant plants were identified by PCR of genome DNA with LP, RP and LB specific primers. **(C)** Semi-quantitative RT-PCR analysis of *AtWSD1* transcript levels in leaves of *wsd1* mutant and wild-type (WT) Arabidopsis plants. The Arabidopsis *AtActin2* (At3g18780) was used as an internal control.

**Supplementary Figure S2 |** Phylogenetic tree of WSD enzymes from sunflower (HaWSD) and Arabidopsis (AtWSD). The neighbor-joining (NJ) tree was generated using the MEGA 7 software with 1,000 bootstrap replicates.

**Supplementary Table S1 |** Primer sequences used for qRT-PCR*, HaWSD9* cloning, vector construction, and transgenic confirmation.

**Supplementary Table S2 |** The information of sunflower HaWSD and Arabidopsis AtWSD sequences in this study.
